# Role of laparoscopy in peritonitis

**Published:** 2013

**Authors:** Ahmed Khan Sangrasi, K. Altaf Hussain Talpu, Nandlal Kella, Abdul Aziz Laghari, Mujeeb Rehman Abbasi, Jawaid Naeem Qureshi

**Affiliations:** 1 **Ahmed Khan Sangrasi, FCPS, Liaquat University of Medical & Health Sciences Jamshoro, Sindh, Pakistan.**; 2 **K. Altaf Hussain Talpur, FCPS, Liaquat University of Medical & Health Sciences Jamshoro, Sindh, Pakistan.**; 3 **Nandlal Kella, FCPS, Liaquat University of Medical & Health Sciences Jamshoro, Sindh, Pakistan.**; 4 **Abdul Aziz Laghari, FRCS, Liaquat University of Medical & Health Sciences Jamshoro, Sindh, Pakistan.**; 5 **Mujeeb Rehman Abbasi, FRCS, Liaquat University of Medical & Health Sciences Jamshoro, Sindh, Pakistan.**; 6 **Jawaid Naeem Qureshi, FRCS, Liaquat University of Medical & Health Sciences Jamshoro, Sindh, Pakistan.**

**Keywords:** Peritonitis, Laparoscopy, Management

## Abstract

***Objective: ***Laparoscopy has gained clinical acceptance in many subspecialties in the last decade. The conventional open surgery for peritonitis carries significant morbidity and mortality. The present study was done to extend and evaluate benefits of minimally invasive surgery in this subset of patients.

***Methods: ***This was a prospective study spanning over a period of four years. All those patients diagnosed as having peritonitis on clinical assessment and preoperative investigations and those who were stable enough haemodynamically were included in this study. After initial resuscitation for few hours, they underwent diagnostic and therapeutic laparoscopy to identify the cause of peritonitis and to confirm the pathology. All cases were done under general anesthesia, using three standard ports at appropriate sites according to pathology. Patients were treated by different procedures either laparoscopically or with laparoscopic assistance after diagnosis. Operative and post operative data was collected and analyzed.

***Results: ***Ninety two cases of peritonitis underwent diagnostic and therapeutic laparoscopy. Mean age of patient was 46.5 years. 24 patients were diagnosed as perforated duodenal, in 14 (58.3%) patients laparoscopic suture repair was done and in 8 (33.3%) small upper midline incision was given and perforation was repaired. Out of 32 patients having perforated appendix, 25 (78.1%) patients laparoscopic appendectomy was done while in 7 (21.8%) perforation was dealt by laparoscopic assistance. Out of 14 patients of ileal perforation 6 (42.8%) with minimal contamination laparoscopic suture was applied, while in 8 (57.1%), perforated loop was brought out by making small window and perforation was closed. All 22 patients with pelvic sepsis needed only aspiration of pus and peritoneal lavage. Only one patient died post operatively and 2 (2.1%) patients developed fistula. 6 (6.5%) patients developed port site infection.

***Conclusion: ***Laparoscopic management is feasible, safe and effective surgical option for patients with peritonitis due to different abdominal emergencies in properly selected cases with higher diagnostic yield and a faster postoperative recovery.

## INTRODUCTION

Peritonitis is quite common cause of surgical emergency and surgical intervention. The increased acceptance of laparoscopy due to its proven benefits of less pain, short hospitalization and decreased morbidity^1^^-^^4^ has encouraged surgeons to use it where it was previously considered as relatively contraindicated.

Laparoscopy has been used for gastrointestinal diseases like perforated peptic ulcer and colonic perforations as early as 1990’s.^2^^,^^3^^,^^5^^-^^10^ Besides its established role in elective upper gastrointestinal surgery, it is being attempted in patients with generalized peritonitis and accompanying severe physiological disturbances.^11^^,^^12^ Many published reports have shown that laparoscopic peritoneal lavage can be effectively performed and perforations can be closed safely.^7^^,^^9^^,^^10^^,^^13^^,^^14^ Encouraged by these studies we conducted present study to extend benefit of minimal invasive surgery in gastrointestinal diseases with localized or generalized peritonitis.

## METHODS

This was a prospective study over a period of four years conducted in department of surgery, Liaquat University of Medical and Health Sciences Jamshoro from July 2008 to June 2012. All those patients who were diagnosed as having peritonitis and were stable haemodynamically were included in the study. We excluded the patients with obvious mechanical obstruction of small and large bowel, chronic recurrent small bowel obstruction and peritonitis due to colonic perforations. As this was our initial experience to use laparoscopy in peritonitis we did not include the patients with previous abdominal surgery, patients with severe sepsis, too ill to withstand pneumoperitoneum. Patients in shock or having major ileus with massive bowel distention were also not considered for laparoscopy.

All patients underwent base line investigations like complete blood picture, serum urea and creatinine level, ultrasound, chest x-rays, plain x-ray abdomen and electrocardiogram (ECG). Patients were resuscitated for initial few hours with intravenous fluids. Electrolytes were corrected and parentral anti-biotics (cefurozime and metronidazole) were instituted. All those patients who were found anemic, blood transfusions were given. These patients underwent either emergency (within first 12 hours) or urgent (between the first 12 to 24 hours) abdominal surgical operations. Inclusion criteria of patients were on clinical grounds depending on the clinical findings and preoperative investigations. Laparoscopy was then performed to identify the cause of pathology and to confirm the diagnosis by finding purulent fluid in the peritoneal cavity. The diagnosis of peritonitis was based on laparoscopic findings of purulent fluid in peritoneal cavity.

This was a non randomized study; consent was taken from all patients after detailed counseling regarding all pros and cons of surgery. A specialized proforma was designed and operative and postoperative variables were recorded and data was analyzed.

***Technique: ***All operations were done in supine position under general anesthesia and carbon dioxide was used for creation of pneumoperitoneum. Bladder catheterization was carried out in all patients and naso-gastric suction drainage tube was placed in patients according to nature of the disease. First access port was placed with an open technique. Peritoneal cavity was explored after introduction of optical system through 10-mm umbilical port. Further operating ports were placed according to the nature of the disease. In the presence of diffused peritonitis, the first step was to evacuate purulent peritoneal collections and thorough irrigation of peritoneal cavity with isotonic warm saline was performed by irrigation-suction device. Diagnostic laparoscopy was then done to identify the causative pathology and establish the diagnosis. Patients were then managed either by laparoscopically or underwent to a conversion for a open operation. Diagnostic accuracy and results of therapeutic laparoscopic procedures were evaluated according to the origin of the peritonitis.

## RESULTS

During the study period 138 patients (85 Male, 53 Female) with mean age of 46.5 yrs range (13-65) underwent laparoscopy. Peritonitis was present in 92 (66.6%) cases. The exact cause of peritonitis was established in 88 percent (81 of 92) by laparoscopy. Conversion to laparotomy was required in 17 patients to clarify the diagnosis suspected at laparoscopy as shown in Table-I. By laparoscopic exploration clinical pre-operative diagnosis was changed in 31 (22.4%) cases, therefore an unnecessary laparotomy was avoided in 10 (7.2%) of these patients.

**Table-I:** Causes of peritonitis and diagnostic results

*Aetiology*	*Total*	*Correct Laparoscopic Diagnosis*	*Conversions (%)*	*Average Operative Time (mins)*	*Mean Hospital Stay (Days)*	*Complications (%)*
Perforated appendicitis	32	29	7 (21.8)	85	7	2 (6.2)
Perforated gastroduodenal ulcer	24	19	8 (33.3)	110	12	3 (12.5)
Small Bowel perforation	14	11	4 (28.5)	70	9	2 (14.2)
Pelvic sepsis	21	21	-	55	7	2
Primary peritonitis	1	1	-	60	8	-

The appendicular localized or generalized peritonitis was present in 32 cases with mean age of 35 (range 12-75) years. Mean operative time was 85 (range 40-150) minutes. Seven cases (21.8%) required either laparoscopic assistance or conversion to open surgery. In majority of these cases conversion was related to difficulty in dissection. 25 patients underwent laparoscopic appendectomy. There was no operative or post operative mortality, mean hospital stay was 7 days. Morbidity rate was recorded as 6.2 percent (2 of 32). These 2 patients developed wound infection postoperatively. Appendicular peritonitis in these cases was due to perforated appendix, one developed port site infection and second surgical site infection postoperatively.

Twenty four patients were admitted with perforated gastro-duodenal ulcer. Mean age of patients was 48 (range 22-75) years. In majority of these patients (83%) suture closure of the perforation was performed. In the rest of patients (17%) suture closure with omentoplasty was performed. Mean operative time recorded was 110 (range, 60-170) minutes. The conversion rate in this subset of patients was 33.3 percent (8 of 24) patients. In 8 cases reason for conversion was inability to locate the site of perforation. The mean hospital stay was 12 days. One patient of gastro-duodenal perforation died postoperatively with mortality rate of 4 percent. This high risk patient was old age (75 years) and died from cardiac failure postoperatively. The morbidity rate was 12.5 percent.

Fourteen patients were admitted to our unit with suspected small bowel perforation, with mean age of 35 (range, 14-65) years. All of these patients presented with peritonitis (either localized or generalized). The average operative time recorded was 55 (range, 45-90) minutes. In 10 cases (71%) suture closure was performed. In 4 cases procedure was converted to open surgery because of difficulty to identify perforation in 2 cases and 2 cases required resection due to ischaemia.

Miscellaneous causes of peritonitis are presented in Table-I. Twenty one patients of pelvic sepsis and one patient of primary peritonitis underwent laparoscopic management; there was no conversion in this group. One patient developed postoperative intra-abdominal sepsis and other subphrenic abscess.

## DISCUSSION

Laparoscopic management has become the preferred modality for various surgical diseases due to the possibility of correctly diagnosing in treating them at the same time.^15-21^ Peritonitis is quite common cause of acute abdomen in general surgical wards. Historically, exploratory laparotomy has been the main stay of the treatment in patients with acute abdomen. Laparoscopic management has previously been considered as a relative contraindication for acute small bowel obstruction and peritonitis. Peritonitis is still considered as a contraindication to laparoscopic approach because of the theoretical risk of enhanced bacteremia and endotoxemia by pneumoperitoneum.^15^^,^^17^^,^^21^ After gaining wide acceptance in many fields, the limitations of laparoscopy are decreasing rapidly. Now many surgeons are attempting to use it in areas where previously it was considered as contraindicated.

Recent studies have shown its use in repair of small bowel perforation,^22^ perforated peptic ulcers^23^ and peritonitis due to various other abdominal emergencies.^24^^,^^25^ We conducted this study to assess the safety and usefulness of laparoscopy as a diagnostic and therapeutic tool in the management of peritonitis. The feasibility, safety and benefits of minimally invasive surgery over the conventional open surgical procedures were considered. Diagnostic accuracy of laparoscopic exploration is reported to be around 90 percent^26^^,^^27^, but as higher as 98% as reported by Kirshtein.^25^ We obtained correct diagnosis in slight lower percentage (88%) in comparison to these studies.

However our diagnostic yield is slightly better than as reported by Navez et al*.*^27^ This may be due to the reason that this study included cases of acute peritonitits, furthermore laparoscopic diagnosis was considered to be correct only if the exact origin of pathology causing peritonitis was identified. In acute peritonitis thorough exploration of the abdominal cavity is quite difficult due to inflammation and bowel distention. Peritoneal hyperaemia also diminishes the quality of the image due to absorption of light. Most of the conversions in this study were due to the incomplete diagnosis, while rest of these was due to therapeutic reasons. Due to poverty, illiteracy and unavailability of medical facilities in rural areas in this part of world presentation of patients is comparatively late. Therefore many patients present with peritonitis due to appendicitis and gastroduodenal perforations. Feasibility and safety of laparoscopic treatment was demonstrated in these patients. Majority of the patients underwent laparoscopic appendectomy successfully, without any mortality, major operative and postoperative complication. Seven patients (22 percent) with appendicitis required conversion to open surgery. In three patients appendix was gangrenous and perforated, while in rest of four patients there was difficulty in dissection. Conversion rate in this study is equivalent or slightly better than few other studies.^27^ Mean hospital stay in cases of appendicular peritonitis in this study was 7 days which is almost in consistence with similar study by Navez B et al^24^ showing slightly longer postoperative stay of 8 days. Morbidity and mortality in our study was 6.2% and zero respectively, versus 9% and 1% respectively by Navez B et al.

There are many studies showing encouraging results of laparoscopic management in perforated peptic ulcer disease^23^^,^^28^, others recommend routine use of laparoscopic repair for perforated peptic ulcer.^29^ We treated 16 patients successfully by laparoscopic approach and required conversion to open surgery in eight (33 percent) cases. Our conversion rates in this group are higher than other studies reported by Navez et al and Ferdinando et al reporting conversion rates of 4 and 12 percent respectively.^27^^,^^30^ This increased rate of conversion may be due to our initial experience of using laparoscopy for managing peritonitis and should improve by increasing learning curve. Mean hospital stay in perforated gastroduodenal ulcers in this study was 12 days, which is comparatively longer than studies conducted by Minutolo V et al^28^ and Robertson GS et al^23^ which shows mean hospital stay as 5.8 days and 5 days respectively. But this difference is due to reluctance of patients in this part of world to discharge earlier in spite of feeling well. In this study morbidity in gastro-duodenal ulcers was higher than the study by Minutolo V et al (12.5% vs. 2.56%). This increased morbidity may be attributed to late presentation of patients in third world countries due to poverty, ignorance and lack of medical facilities. We had a comparable mortality of zero in both studies. Small bowel perforations are not uncommon in third world countries because of increased prevalence of typhoid and tuberculosis disease involving small gut. With successful and encouraging results of laparoscopic management of peritonitis, we demonstrated this approach of in patients with bowel perforation.

We successfully managed 10 (71 percent) patients by laparoscopic approach by simple suture closure without any mortality or major complication. Our results in this group are in consistence with similar study by Rajeev Sinha et al^22^ reporting successful intra-corporeal suturing for small bowel perforation. Average postoperative hospital stay in study by Rajeev Sinha varied between 7-10 days while in our study it was 9 days which are almost in consistence with each other. Similarly morbidity in our study was 14.12% vs. 8% in study by Rajeev Sinha et al. There was no mortality in both studies. In primary peritonitis suction of intra-peritoneal pus and cleaning of the abdominal cavity by irrigation-suction device can be achieved more accurately under laparoscopic guidance. However copious use of saline to lavage peritoneal cavity should be carried out cautiously in localized intra-abdominal abscess because at least theoretically it has some disadvantages.^25^

In Intra-abdominal sepsis and peritonitis the role of laparoscopy is mainly diagnostic and we found good results of laparoscopy in pelvic sepsis and primary peritonitis. In these patients diagnostic yield was 100 percent and no conversion was required. With the laparoscopic devices used currently, pancreas, posterior aspect of gastric wall and retro-peritoneum cannot be visualized. Therefore any suspected pathology in these areas, conversion to open surgery by laparotomy will be the best approach. By our experience we found laparoscopic management of peritonitis in abdominal emergencies feasible with acceptable morbidity and mortality. This morbidity and mortality is comparable with open surgery as reported by some studies^31^, but this might be due to our patient’s selection criteria.

## CONCLUSION

Laparoscopic management is feasible, safe and effective surgical option for patients with peritonitis due to different abdominal emergencies in properly selected cases with higher diagnostic yield and a faster postoperative recovery.

## References

[1] Druart ML, Van-Hee R, Etienne J, Cadiere GB, Gigot JF, Legrand M (1997). Laparoscopic repair of perforated duodenal ulcer. A prospective multicenter clinical trial. Surg Endosc.

[2] Katkhouda N, Mavor E, Mason RJ, Campos GM, Soroushyari A, Berne TV (1999). Laparoscopic repair of perforated duodenal ulcers: outcome and efficacy in 30 consecutive patients. Arch Surg.

[3] Lau WY, Leung KL, Kwong KH, Davey IC, Robertson C, Dawson JJ (1996). A randomized study comparing laparoscopic versus open repair of perforated peptic ulcer using suture or sutureless technique. Ann Surg.

[4] So JB, Kum CK, Fernandes ML, Goh P (1996). Comparison between laparoscopic and conventional omental patch repair for perforated duodenal ulcer. Surg Endosc.

[5] Laws HL, McKernan JB (1993). Endoscopic management of peptic ulcer disease. Ann Surg.

[6] Katkhouda N, Mouiel J (1991). A new technique of surgical treatment of chronic duodenal ulcer without laparotomy by videocoelioscopy. Am J Surg.

[7] Lau WY, Leung KL, Zhu XL, Lam YH, Chung SC, Li AK (1995). Laparoscopic repair of perforated peptic ulcer. Br J Surg.

[8] Lee PY, Lang KL, Lai PB, Lau JW (2001). Selection of patients for lap repair of perforated Peptic ulcer. Br J Surg.

[9] Mouret P, François Y, Vignal J, Barth X, Lombard-Platet R (1990). Laparoscopic treatment of perforated peptic ulcer. Br J Surg.

[10] Nathanson LK, Easter DW, Cuschieri A (1990). Laparoscopic repair/peritoneal toilet of perforated duodenal ulcer. Surg Endosc.

[11] Schein M, Gecelter G, Freinkel Z, Gerding H (1990). APACHE II in emergency operations for perforated ulcers. Am J Surg.

[12] Boey J, Wong J, Ong GB (1982). A prospective study of operative risk factors in perforated duodenal ulcers. Ann Surg.

[13] Perrissat J, Collet D, Edge M (1992). Therapeutic laparoscopy. Endoscopy.

[14] Beriot J, Champault GG, Labhar E (1992). Sutureless laparoscopic treatment of perforated duodenal ulcer. Br J Surg.

[15] Agresta F, De Simone P, Bedin N (2004). The laparoscopic approach in abdominal emergencies: a single-center 10-year experience. JSLS.

[16] Agresta F, Piazza A, Michelet I, Bedin N, Sartori CA (2000). Small bowel obstruction. Laparoscopic approach. Surg Endosc..

[17] Agresta F, Michelet I, Coluci G, Bedin N (2000). Emergency laparoscopy: a community hospital experience. Surg Endosc.

[18] Branicki FJ (2002). Abdominal emergencies: diagnostic and therapeutic laparoscopy. Surg Infect (Larchmt).

[19] Cuschieri A (1998). Cost efficacy of laparoscopic vs open surgery. Hospitals vs community. Surg Endosc.

[20] Lau WY, Fan ST, Yiu TF, Chu KW, Suen HC, Wong KK (1986). The clinical significance of routine histopathologic study of the resected appendix and safety of appendiceal inversion. Surg Gynecol Obstet.

[21] Sauerland S, Agresta F, Bergamaschi R, Borzellino G, Budzynski A, Champault G (2006). Laparoscopy for abdominal emergencies: evidence-based guidelines of the European Association for Endoscopic Surgery. Surg Endosc.

[22] Sinha R, Sharma N, Joshi M (2005). Laparoscopic repair of small bowel perforation. JSLS.

[23] Robertson GS, Wemyss-Holden SA, Maddern GJ (2000). Laparoscopic repair of perforated peptic ulcers. The role of laparoscopy in generalised peritonitis. Ann R Coll Surg Engl.

[24] Navez B, Tassetti V, Scohy JJ, Mutter D, Guiot P, Evrard S (1998). Laparoscopic management of acute peritonitis. Br J Surg.

[25] Kirshtein B, Roy-Shapira A, Lantsberg L, Mandel S, Avinoach E, Mizrahi S (2003). The use of laparoscopy in abdominal emergencies. Surg Endosc.

[26] Nagy AG, James D (1989). Diagnostic laparoscopy. Am J Surg.

[27] Navez B, d'Udekem Y, Cambier E, Richir C, de Pierpont B, Guiot P (1995). Laparoscopy for management of nontraumatic acute abdomen. World J Surg.

[28] Minutolo V, Gagliano G, Rinzivillo C, Minutolo O, Carnazza M, Racalbuto A (2009). Laparoscopic surgical treatment of perforated duodenal ulcer. Chir Ital.

[29] Siu WT, Chau CH, Law BK, Tang CN, Ha PY, Li MK (2004). Routine use of laparoscopic repair for perforated peptic ulcer. Br J Surg.

[30] Agresta F, Ciardo LF, Mazzarolo G, Michelet I, Orsi G, Trentin G (2006). Peritonitis: Laparoscopic Approach. World J Emergency Surg..

[31] Bailey IS, Rhodes M, O’Rourke N, Nathanson L, Fielding G (1998). Laparoscopic management of acute small bowel obstruction. Br J Surg..

